# Assessment of the Hazards Occurring During the Thermal Decomposition and Combustion Process in a Toothed Belt Transmission

**DOI:** 10.3390/ma18071637

**Published:** 2025-04-03

**Authors:** Łukasz Warguła, Piotr Kaczmarzyk, Bartosz Wieczorek, Daniel Małozięć, Anna Dziechciarz

**Affiliations:** 1 Institute of Machine Design, Faculty of Mechanical Engineering, Poznan University of Technology, Piotrowo 3, 60-965 Poznań, Poland; bartosz.wieczorek@put.poznan.pl; 2 Scientific and Research Centre for Fire Protection, National Research Institute, Nadwiślańska 213, 05-420 Józefów, Poland; pkaczmarzyk@cnbop.pl (P.K.); dmaloziec@cnbop.pl (D.M.); adziechciarz@cnbop.pl (A.D.)

**Keywords:** toxicometric indicators (W_LC50SM_), machine fires, synchronous transmissions, fire hazard, toxic smoke

## Abstract

This article demonstrates that machine fires caused by a belt transmission are a fundamental and current research problem. The aim of this work is to identify the hazards during thermal decomposition and combustion of a transmission with a toothed belt, used as a drive or conveyor belt to synchronise mechanisms. The analysis distinguished belts in a polyurethane or rubber cushion with a Kevlar, steel, or polyurethane cord. The belts’ composite structure can be a source of unpredictable emissions and toxic substances of varying concentrations and compositions during thermal decomposition and combustion. To evaluate the compared belts, a testing methodology was used to determine the toxicometric indicators (W_LC50SM_), according to which it was possible to assess the toxicity of the thermal decomposition and combustion products following EU standards. The analysis was carried out based on the recorded emissions of chemical compounds during the thermal decomposition and combustion of polymer materials at three different temperatures (450, 550, and 750 °C). The least favourable toxicometric indicators (W_LC50SM_) are found in rubber cushion belts, which are very toxic (about 13 g/m^3^) and toxic (about 40 g/m^3^) materials. The results show that thermoplastic polyurethane cushion belts are moderately toxic materials, with a W_LC50SM_ index ranging from 411 g/m^3^ to 598 g/m^3^.

## 1. Introduction

The thermal decomposition products resulting from plastic fires pose the greatest risk in a fire. Industrial infrastructure and machinery fires remain a significant and persistent problem. In 2019, Val-Aguasca et al. showed that 19% of the causes of fire in combine harvesters were due to transmission and conveyor belts with defective bearings [1]. In 2020, Tomašková et al. stated that dry matter (straw, hay) presses with closed working chambers posed the most significant fire risk in agricultural machinery [2]. The most common cause of fire in agricultural balers is defective tension and control rollers, which are responsible for the operation of packing belts [2]. Transporting flammable materials using conveyor belts is also a major fire hazard. Damaged conveyor rollers [3,4] that come into contact with the conveyor belt can cause a fire, a problem that has been widely described in coal transport [5,6,7]. Fires involving belts result from a combination of mechanical, chemical, and electrical factors. The most common causes include friction and overheating due to seized rollers or improper belt alignment [8,9,10], electrical equipment failures [11,12], and direct exposure to fire [11]. Studies are being carried out on fire protection and fire mitigation. These studies are related to rapid fire detection and extinguishing systems, pre-fire prevention systems, or the research and application of fire-resistant materials or materials with limited adverse effects under fire conditions. Belts are subjected to fire safety tests, which assume they will not ignite and should have limited fire spread if ignited. To assess these properties, belts are subjected to tests mapping the causes of fire, such as sliding friction due to roller blockage and the movement of the belt or vice versa; ignition of the transported material; ignition due to the fire spreading in the belt area; or an electrical resistance test, which mimics the possibility of the accumulation and subsequent discharge of a static electrical charge on moving conveyors [13]. There has been a noticeable increase in the materials used for conveyors or drive belts. These mainly include polymer composites with a matrix of polyamide or polyurethane, SBR or NBR rubber, rubber, steel, and natural materials such as leather or fabric [14,15,16]. Their properties have been tested for many aspects, such as mechanical strength or machinability [14,15,16]. Additionally, while research on V-belts [17] and flat belts [18,19] was carried out by the authors and described in earlier works, there are no studies in the literature on transport and drive belts regarding the type and amount of emissions of chemical compounds formed during the thermal decomposition and combustion of toothed belts. Investigations of machine parts in transport or transmission systems that emit toxic or hazardous chemical compounds in exhaust gases during thermal decomposition and combustion provide essential information for fire detection system designers based on changes in the air composition in the machine or production belt under investigation. The difficulty of such an analysis is that some transport materials may also emit chemical compounds into the atmosphere, similar to how carbon monoxide (CO) and hydrocarbons (HCs) are released during a fire (e.g., coal and biomass transport) [20,21]. Knowledge of the toxic compounds emitted during the thermal decomposition and combustion of conveyor and drive dogs is essential information for rescuers during a rescue operation. This information is also important for paramedics assisting people who have been removed from the hazard, as exhaust fumes can poison them. In many designs of material handling systems, the conveyor belt is the main object of fire, as the rest of the structure is steel. Available methods for recognising a fire or for recognising symptoms that may contribute to fire are thermal detection [3,4], video detection [22], vibration detection with recognition of damaged rollers [23], or gas fire detection systems [24]. There are studies in the literature on chemical emissions from the thermal decomposition and combustion of V-belts [17] used as drive belts and overload couplings, which can reach high temperatures and are a source of fire under select operating conditions [25]. Flat belts [18,19], which are used as conveyor belts or drive belts with the possibility of acting as an overload coupling, have also been investigated. Toothed belts differ significantly in their material composition, structural design, and functional performance, which can impact their thermal degradation behaviour [26,27,28].

This paper presents studies on chemical emissions from the thermal decomposition and combustion of toothed belts, which act as conveyor or drive belts characterised by ensuring the synchronised movement of the machine system. Toothed belts differ in structure from flat and V-belts in that they transmit speed and torque not only frictionally, as flat and V-belts, but primarily by shape [29].

This paper details the toxicometric indicators (W_LC50M_) that were determined with the material classification according to the thermal decomposition and combustion products concerning their toxicity. The results of the air composition study in the initial stage of the fire are presented as a guideline for experts for determining the cause of a fire in the area where a continuous air composition monitoring system is located. The study also aims to provide information about the nature of the hazard for rescuers taking part in a firefighting operation, the type of poisoning of the occupants in the fire area for paramedics, and the kinds of chemical compounds that enable their identification for designers of fire recognition systems.

## 2. Materials and Methods

### 2.1. Materials

**Figure 1:**
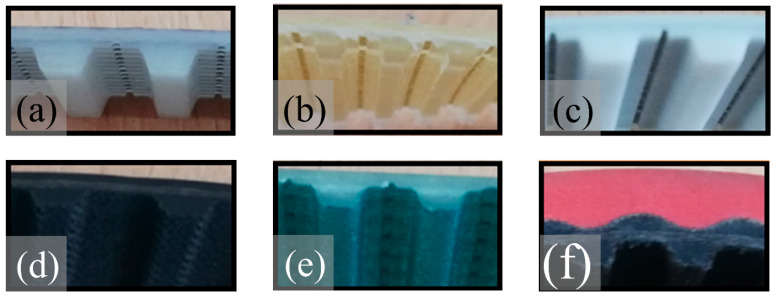
Toothed belts: (**a**) P1, (**b**) P2, (**c**) P3, (**d**) P4, (**e**) P5, and (**f**) P6.

Six types of commercial toothed belts were analysed to determine the toxicometric indicators. Such belts are used as conveyor or drive belts characterised by maintaining the synchronous motion of the driven mechanisms. Comparisons were made between belts made of different materials, such as a classic toothed belt with a polyurethane matrix (with the trade name Vulcolan) and a steel cord (Synchroflex T10), from Continental ContiTech (Hanover, Germany), designated as belt P1. Another polyurethane belt tested (with the trade name Desmopan) with a Kevlar cord was the Breco VT5 belt from BRECOflex Co., LLC (Eatontown, NJ, USA) designated as belt P2. The P3 toothed belt was a Breco VT10 belt in a polyurethane matrix (trade name Desmopan) with a steel cord from BRECOflex Co., LLC (Eatontown, NJ, USA). The other belts tested, P4, P5, and P6, were belts whose teeth were additionally covered with a vulcanised fabric. The H-type toothed belt in a synthetic rubber matrix with a polyester cord and a tooth cover of vulcanised nylon fabric from Optibelt GmbH (Höxter, Germany) is designated as P4. The Eltach T10 belt was a polyurethane belt with a Kevlar cord with teeth covered with polyamide fabric from Elatech Technology in Motion (Val Brembilla, Italy), designated as belt P5. The belt, designated P6 (T10), was characterised by its complex construction. The belt matrix was made of polyurethane on the outside (red colour). In contrast, the inside (teeth) was made of rubber (black colour), the teeth were covered with a vulcanised nylon fabric cover, and the cord was made of polyester by Wilhelm Herm Müller Polska Sp. z o.o. (Bydgoszcz, Poland). The tested toothed belts are shown in Figure 1.

### 2.2. Methods

To evaluate the toxic emissions from thermal decomposition and combustion, Fourier-transform infrared spectroscopy (FTIR) was employed. The analysis was conducted using a Jasco FT-IR 4700 spectrometer (Tokyo, Japan), operating in the spectral range of 4000–400 cm^−1^ with a resolution of 4 cm^−1^ and an average of 16 scans per measurement. The Jasco FT-IR 4700 spectrometer offers high accuracy and precision, with a wavenumber accuracy of ±0.01 cm^−1^, a maximum resolution of 0.4 cm^−1^, and a signal-to-noise ratio exceeding 35,000:1. These characteristics make it well suited for applications requiring high resolution, such as gas analysis. The gas analyser was calibrated using reference gases with known concentrations. The collected spectral data were processed using Spectra Manager software (ver. 2, Jasco, Easton, MD, USA). The methodology for measuring the toxicity of the thermal decomposition and combustion products was based on a quantitative chemical analysis of the emitted compounds. Due to the lack of dedicated standards for assessing toxic emissions from machine parts, a fire safety testing standard typically used in construction was applied. Approximately 4.0 ± 0.1 g of each belt sample was prepared, ensuring the uniform distribution of representative material layers. The samples were weighed using the AS 220.R2 PLUS analytical balance from Radwag Wagi Elektroniczne (Radom, Poland), featuring a readability of 0.1 mg and a maximum capacity of 220 g. The tests followed the Polish standard PN-B-02855:1988 [30], which outlines procedures for assessing fire hazards and aligns with internationally recognised standards, including the French NFx70-100 (1986) [31], the German DIN 53.436 [32], and the Russian GOST 12.1.044-89 [33]. The study involved controlled thermal decomposition at three temperatures: 450, 550, and 750 °C, with an air supply rate of 100 ± 10 dm^3^/h in a countercurrent flow setup. The measurement uncertainty of the temperature, according to the calibration certificate of the measurement system, was 2.1 °C. The flow rate was regulated and maintained using a rotameter RTU-06-300; the measurement uncertainty according to the calibration certificate was 1 dm^3^/h (Z.A. “Rotametr Sp. z o.o.”, Gliwice, Poland). The selection of 450 °C, 550 °C, and 750 °C follows the requirements of this standard, ensuring the results are comparable to other studies using the same methodology. These temperatures are commonly used in fire testing because they represent different stages of material degradation in real-world scenarios. First, 450 °C represents the early stages of decomposition, where materials begin to break down and release volatile compounds. Second, 550 °C corresponds to more advanced degradation, where significant thermal decomposition occurs, producing toxic gases and particulates. Third, 750 °C simulates conditions in fully developed fires, where materials burn intensely, leading to maximum gas emissions and residue formation. While actual machine fires can vary in temperature depending on factors such as fuel sources and ventilation, these standardised temperatures provide a controlled and reproducible framework for assessing the fire behaviour and toxicity of materials.

The testing was conducted in two stages. The first stage involved qualitative analysis to identify the composition of the thermal decomposition and combustion products by comparing the obtained spectra with the reference spectra. The analysis aimed to detect the presence of carbon (C), hydrogen (H), nitrogen (N), sulphur (S), and chlorine (Cl), ensuring the identification of potential emissions such as carbon monoxide (CO), carbon dioxide (CO_2_), hydrogen cyanide (HCN), nitrogen dioxide (NO_2_), hydrogen chloride (HCl), and sulphur dioxide (SO_2_). Compounds that were not detected in the qualitative analysis were excluded from the subsequent quantitative assessment. In the second stage, quantitative analysis was conducted to determine the emissions of carbon monoxide (CO), carbon dioxide (CO_2_), hydrogen cyanide (HCN), nitrogen dioxide (NO_2_), hydrogen chloride (HCl), and sulphur dioxide (SO_2_) during thermal decomposition and combustion. The tests were performed at controlled temperatures of 450 ± 5 °C, 550 ± 5 °C, and 750 ± 5 °C, with an airflow rate of 100 ± 10 dm^3^/h. The air was introduced in a countercurrent direction relative to the furnace movement, which proceeded at a rate of 20 mm/min. The complete traversal of the furnace over the sample took 30 min. Gas samples for the quantitative analysis of CO and CO_2_ emissions were collected at intervals of 7.5, 15, and 22.5 min from the start of the test. For compounds such as HCN, NO_2_, HCl, and SO_2_, the emitted gases were captured using absorbing scrubbers throughout the furnace traversal (30 min) and its return to the initial position (5 min). Each test was conducted twice at each temperature, and a third trial was performed if the variation between results exceeded 30% to ensure reliability and reproducibility.

**Figure 2:**
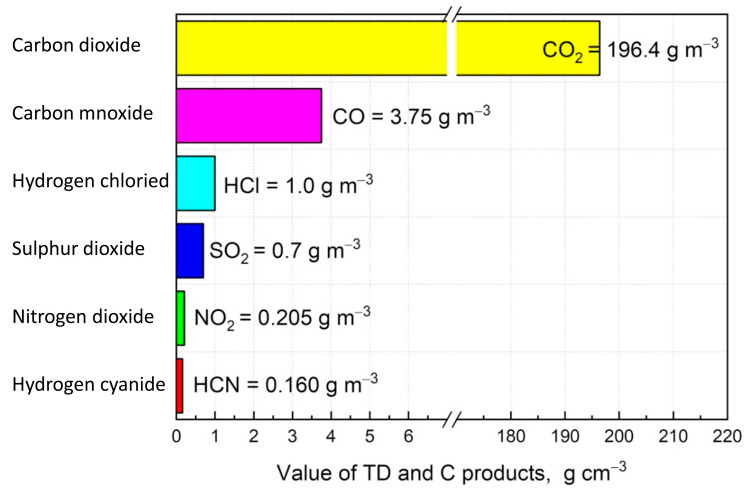
Limit concentration indicators (${LC}_{50}^{30}$) of thermal decomposition and combustion products.

The concentrations of carbon monoxide (CO), carbon dioxide (CO_2_), hydrogen chloride (HCl), hydrogen cyanide (HCN), nitrogen dioxide (NO_2_), and sulphur dioxide (SO_2_) were determined during the tests. The measured concentrations of these gases were recorded and used to calculate their specific emissions (E), which represent the mass of toxic products generated per unit mass of material under controlled test conditions. By correlating these specific emissions (E) with the concentration limits (${LC}_{50}^{30}$), which define the lethal concentration for 50% of the population after 30 min of exposure, the following toxicometric indicators were obtained (Figure 2):

- The toxicometric indicator (W_LC50_) represents the amount of material that, when subjected to thermal decomposition or combustion, generates toxic concentrations of specific decomposition products. It is calculated using Equation (1).

(1) $$W_{LC50}=\frac{{LC}_{50}^{30}}{E},\;g/m^{3}$$

where ${LC}_{50}^{30}$ is the threshold concentration at which 50% of the exposed population is affected within 30 min and is the specific emission of toxic decomposition products (g·g^−1^).

- The toxicometric indicator (W_LC50M_) is derived from the W_LC50_ values for individual thermal decomposition and combustion products at a given temperature, as expressed in Equation (2).

(2) $$\frac{1}{W_{LC50M}}=\sum^{n}\frac{1}{W_{LC50}},$$

where n is the number of samples and ${LC}_{50}^{30}$ corresponds to the lethal concentration for 50% of the population exposed for 30 min (values presented in Figure 2).

- The toxicometric indicator (W_LC50SM_) represents the arithmetic mean of the W_LC50M_ values determined at different temperatures (450 °C, 550 °C, and 750 °C), as shown in Equation (3).

(3) $$W_{LC50SM}=\frac{1}{3}(W_{LC50M\;450{}^{\circ}C}+W_{LC50M\;550{}^{\circ}C}+W_{LC50M\;750{}^{\circ}C})$$

**Figure 3:**
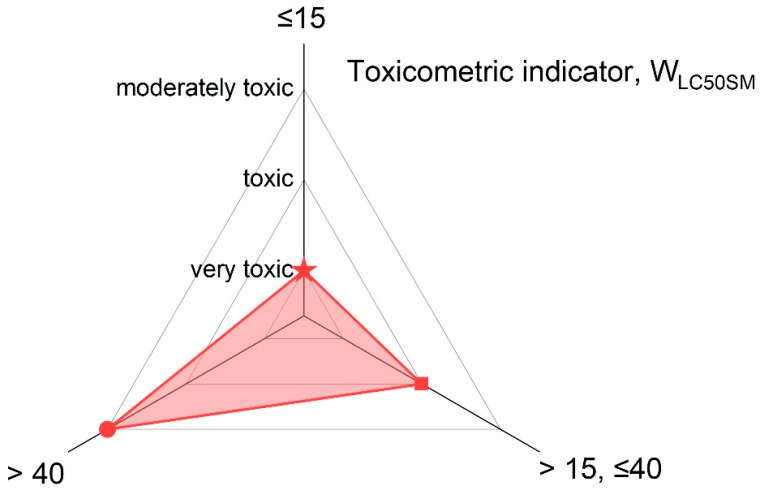
The toxicometric indicator (W_LC50SM_), according to PN-B-02855 [30], for toxic products from the thermal decomposition and combustion of materials.

The main classification criterion for toxic products from the thermal decomposition and combustion of materials is the toxicometric indicator (W_LC50SM_) according to PN-B-02855 [30], which is presented in Figure 3.

The combined measurement uncertainty was determined using the root sum of squares (RSSs) method, incorporating the uncertainty in the FTIR wavenumber (±0.01 cm^−1^), temperature measurement (±2.1 °C), mass measurement (±0.0001 g), and airflow measurement (±1 dm^3^/h). The calculated combined measurement uncertainty was approximately 2.33, considering the respective units of individual uncertainties. The relative uncertainty for each variable was 0.0003% for the FTIR wavenumber accuracy, 0.28% for the temperature measurement, 0.0025% for the sample mass measurement, and 1% for the flow rate measurement.

## 3. Results and Discussion

**Figure 4:**
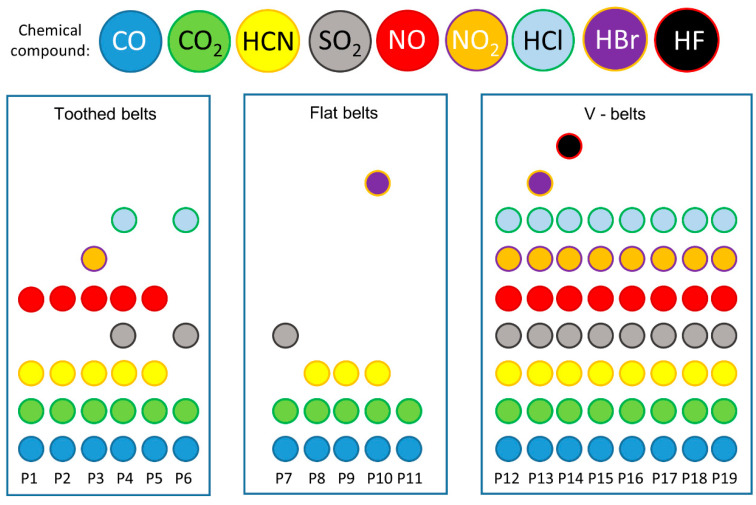
Comparative analysis of toxic emissions generated during the thermal degradation and combustion of toothed and flat belts [18,19] and V-belts [17], where P1–P6 are the toothed belts described in this article, P7–P11 are flat belts described by Krawiec et al. in 2020 [18], and P12–P19 are V-belts described by Krawiec et al. in 2020 [17].

The analysis of the spectra focused on detecting SO_2_, NO_2_, NO, HCN, CO_2_, CO, HCl, HBr, and HF gases in relation to the toxicity assessments. Additionally, a detailed comparison was conducted on the substances identified in the thermal decomposition products of each belt, as presented in Table 1 and Figure 4. It can be observed that the tested belts can emit four to five compounds during thermal decomposition and combustion. All the belts were characterised by emissions of CO and CO_2_, while the emissions of the other compounds differed between the belts depending on the type of materials from which the belts were made. In 2017, Wojtkowiak et al. analysed the perforation processes of conveyor belts, showing, based on research by Sheikh-Ahmad in 2009, that HCN emissions can characterise belts with polyamide (Kevlar) cord during thermal processing methods, e.g., laser cutting, plasma cutting, and EDM (electro-discharging) [15,34]. This is in line with the study’s results, as there were HCN emissions from belts with polyamide cords (P2, P5). It can also be noted that all the belts with a polyurethane matrix (P1, P2, P3, P5) emitted HCN compounds. This is consistent with the results of other researchers who conducted studies on the thermal decomposition and combustion of polymeric materials, e.g., furniture foams showing emissions of CO, CO_2_, HCN, and in some cases HCl, NO_2_, and HF, among others [35,36,37,38]. NO_2_ emissions were shown by only one polyurethane matrix P3 belt, with the trade name Desmopan. In contrast, none of the polyurethane matrix belts showed HCl and HF emissions. It can be noted that SO_2_ and HCl emissions were only characteristic of the thermal decomposition and combustion of rubber belts (P4 and P6). Combustion and thermal decomposition studies of car tyres, which are similar in design and material to belts, showed high CO, CO_2_, SO_2_, and NO_2_ emissions [39,40,41]. In addition, it is possible to notice a coincidence in the emissions of CO, CO_2_, and SO_2_ during the combustion of rubber matrix belts (P4 and P6), as well as no emissions of NO_2_, while emissions of HCl compounds, which are not a characteristic product of rubber combustion, are noticed. It can be surmised that the HCl emissions may originate from the combustion and thermal decomposition of the polyurethane cord used to construct these belts [42], as some types of polyurethane are characterised by emissions of HCl compounds [43,44].

Comparing toothed belts with other types such as V-belts and flat drive belts, depending on the type of conveyor belt, one can see a difference in the number of compounds emitted from the exhaust gas (Figure 2). Emissions from other kinds of belts are taken from the authors’ studies (flat belts [18,19], V-belts [17]). In addition, it can be seen that V-belts had the lowest variation in emitted exhaust compounds. Such belts are characterised by the least variation in material and are most often constructed only of a homogeneous material (polyurethane or rubber) [45,46] or are built with a matrix (polyurethane or rubber) and a cord (steel, Kevlar, or polyester) [47,48]. Toothed belts are more complex, always consisting of a matrix and cords (materials also used in V-belts) [49,50], producing more toxic emissions. In contrast, flat belts used as drive or conveyor belts have the highest number of toxic compounds. These belts often consist of several layers of composites [51,52,53]. The materials used for flat belts are similar to those used for V-belts and toothed belts, but composite layers of natural leather, for example, are also used [18].

It should be emphasised that the results of the experimental studies on the types of emitted compounds represent important and helpful information for paramedics assisting people injured during a fire, who are most often affected by toxic smoke inhalation, which poses a serious threat to health and life. From an application point of view, the results obtained are a source of information for engineers designing fire detection systems, given the types of chemical compounds produced during a fire that need to be monitored in machines equipped with gear belts.

**Figure 5:**
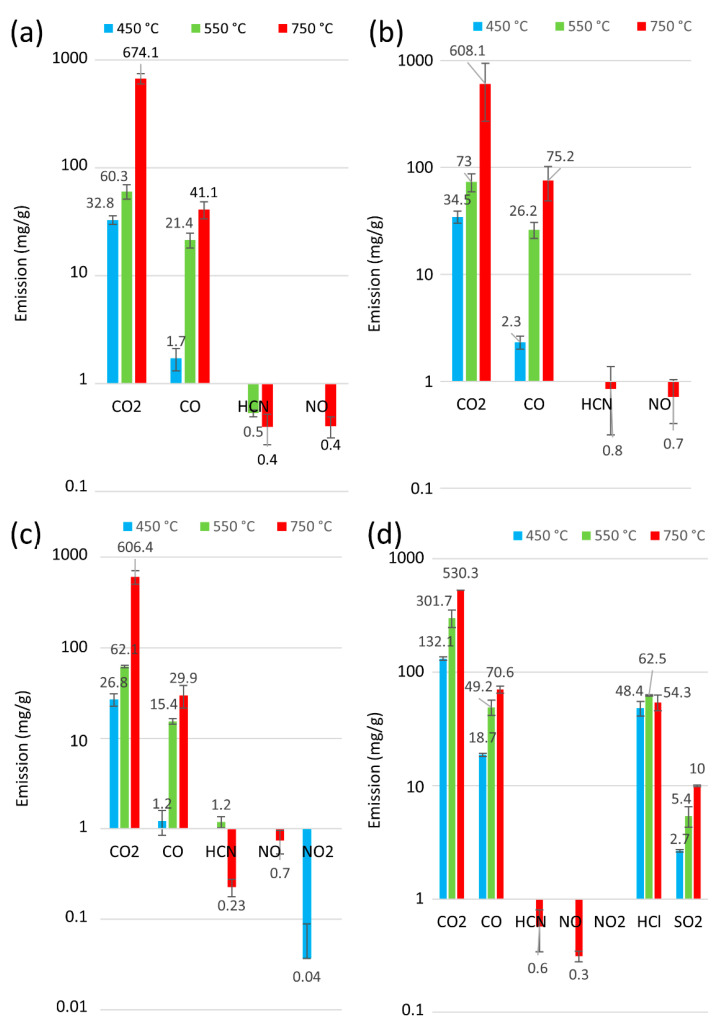
Release of byproducts from the thermal degradation and combustion of toothed belts at 450 °C, 550 °C, and 750 °C: (**a**) P1, (**b**) P2, (**c**) P3, (**d**) P4.

**Figure 6:**
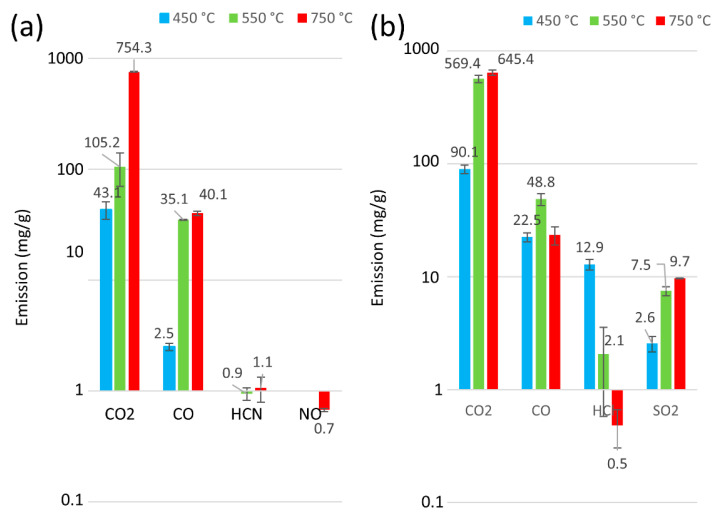
Release of byproducts from the thermal degradation and combustion of toothed belts at 450 °C, 550 °C, and 750 °C: (**a**) P5, (**b**) P6.

**Figure 7:**
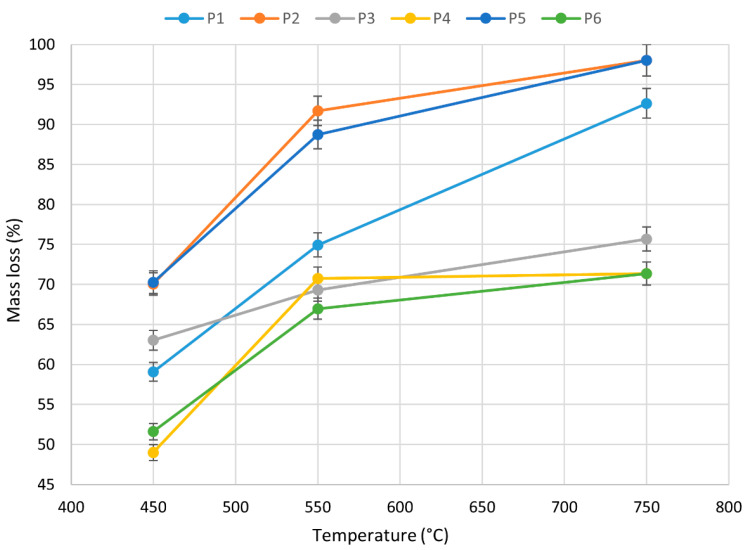
Mass loss of P1–P6 toothed belts undergoing thermal degradation and combustion at 450 °C, 550 °C, and 750 °C.

Information on the type of exhaust compounds emitted in fire detection and neutralisation is essential. However, it is equally important to quantify emissions for the sake of human life and health. The analysis of the number of thermal decomposition products for samples P1 to P6 for 30 min at 450, 550, and 750 °C is presented in Figure 5 and Figure 6. Furthermore, Figure 7 presents an overview of the thermal decomposition and combustion processes, including the mass loss of the tested samples. In general, an increase in CO_2_ emissions was observed for all the belts as the temperature rose. During the initial stage of degradation in a nitrogen environment, CO_2_ was identified as the predominant byproduct for all tested polymers, suggesting the breakdown of urethane bonds [54]. It can be seen that, at 750 °C, all the tested belts had high emissions ranging from 754 mg/g to 530 mg/g. In contrast, at lower temperatures (around 450 °C), the polyurethane matrix belts had CO_2_ emissions ranging from 26 mg/g to 43 mg/g, and the rubber matrix belts showed a more than 50% increase in CO_2_ emissions from 90 mg/g to 132 mg/g, indicating that rubber matrix belts exhibit higher toxicity at lower temperatures. Furthermore, no correlations indicate that the protective fabrics of the teeth in the belts (characteristic of belts P4 to P6) affected the toxicity of these machine parts.

The weight loss of the samples as a function of the thermal decomposition temperature was also recorded. Figure 7 shows that, in general, the weight loss of the belts increased as the temperature increased. It was found that rubber matrix belts (P4 and P6) at the highest measured temperature of 750 °C showed the lowest weight loss of 72 ± 3%, in contrast with polyurethane belts with a steel encoder (P1 and P3). In addition, the study also showed that polyurethane belts with a Kevlar encoder at 750 °C, the highest measurement temperature, had the highest weight loss of 97 ± 2%. The thermal analysis results show that the mass losses of the belts tested were different due to their thermal stability and construction. The lowest mass loss of rubber occurred during its heating at T = 750 °C, due to the relatively high yield of the cross-linking component of the elastomer included in this rubber belt. A similar effect was observed by Slusarski et al. for sealing plates and rubber after heating up to T = 800 °C [55].

The materials’ decomposition rate is mainly due to the type of materials used to manufacture the belts. Many of the belts tested were made of rubber and thermoplastics, i.e., polyamide and polyurethanes, which were reinforced with steel cord or aramid fibre (Kevlar). In the case of nitrile rubber, the initial degradation temperature (the temperature at 5 wt.% mass loss) occurs mainly at about 360 °C [56] and for TPU at about 302 °C [54]. The reinforcement of the polymer matrix used significantly affects the hardness of the belts and their thermal resistance. The rubber belts with cord steel have higher thermal stability and hardness than the TPU belts with Kevlar. In the case of reinforcements, the thermal stability of aramid fibres (Kevlar) is about 520 °C in the air [57], and a steel cord melts above 1100 °C. Aramid fibre’s high resistance to chemicals and high temperatures makes it an ideal component for belts under heavy loads in difficult conditions [58]. Kevlar naturally exhibits flame resistance but can still ignite, with a limiting oxygen index (LOI) of 29. While combustion typically ceases once the ignition source is removed, pulp or dust particles may continue to smoulder after ignition [58]. When Kevlar burns, it generates combustion gases similar to those produced by wool, primarily carbon dioxide, water vapour, and nitrogen oxides. However, under certain conditions, the combustion process may also release carbon monoxide, trace amounts of hydrogen cyanide, and other toxic gases, depending on factors such as the temperature and oxygen availability. For instance, CO_2_ = 1.850 mg/g, CO = 50 mg/g, N_2_O = 14 mg/g, and NH_3_ = 0.5 mg/g of the sample [58]. In the case of polyamide fibre type Nylon 66, the amount of emissions was lower than that of Kevlar, as it was 1.2 mg/g of CO_2_, while the CO emissions were five times higher, at 250 mg/g. In Nylon 66 polyamide fibres, the main combustion product was C_2_H_4_ (50 mg/g), which was not observed in Kevlar.

The release of toxic gases during thermal decomposition and combustion is highly dependent on the temperature, which influences the breakdown of chemical bonds, the oxidation processes, and the generation of new compounds. Higher temperatures generally lead to more complete decomposition and the release of different toxic gases, including hydrogen cyanide (HCN) and nitrogen dioxide (NO_2_). Many synthetic materials, such as polyurethanes, nylons, and certain rubbers, contain nitrogen in their molecular structure. At moderate temperatures, partial degradation occurs, leading to amine compounds and ammonia as initial byproducts. At higher temperatures (above 550 °C), these nitrogen-containing compounds undergo oxidation, favouring the formation of HCN and NO_2_. At lower temperatures, incomplete combustion can lead to the formation of carbon monoxide (CO) and lower concentrations of nitrogen oxides. As the temperature increases, more complete oxidation occurs, resulting in higher emissions of NO_2_, which originate from the oxidation of nitrogen present in both the material and the surrounding air. Hydrogen cyanide is mainly produced from nitrile-containing materials and polyurethanes. At temperatures above 500–600 °C, the thermal degradation of these nitrogen-containing groups releases free cyanide radicals, forming HCN in gaseous form. At higher temperatures (above 700 °C), the oxidation of nitrogen-based compounds and reactions with atmospheric oxygen lead to the formation of nitrogen oxides, particularly NO_2_, which is a highly toxic and irritant gas. The rate of oxidation reactions increases exponentially with the temperature. At 750 °C and above, oxygen availability and thermal energy drive the formation of NO_2_ from NO (produced at lower temperatures), increasing its concentration in fire effluents. In summary, HCN and NO_2_ appear more at higher temperatures due to the progressive breakdown and oxidation of nitrogen-containing materials. HCN is primarily released at mid-to-high temperatures (500–600 °C) due to the thermal decomposition of nitriles and polyurethane-based materials, while NO_2_ formation increases above 700 °C as a result of the oxidation of nitrogen-containing volatiles and atmospheric nitrogen. Higher temperatures lead to more complete oxidation, shifting gas emissions from CO and lower nitrogen compounds toward NO_2_ and other toxic oxidised species. Similar dependencies were observed in studies on similar materials conducted by Rybiński in 2014 [59] and Milczarek and Tarka in 2020 [60].

**Figure 8:**
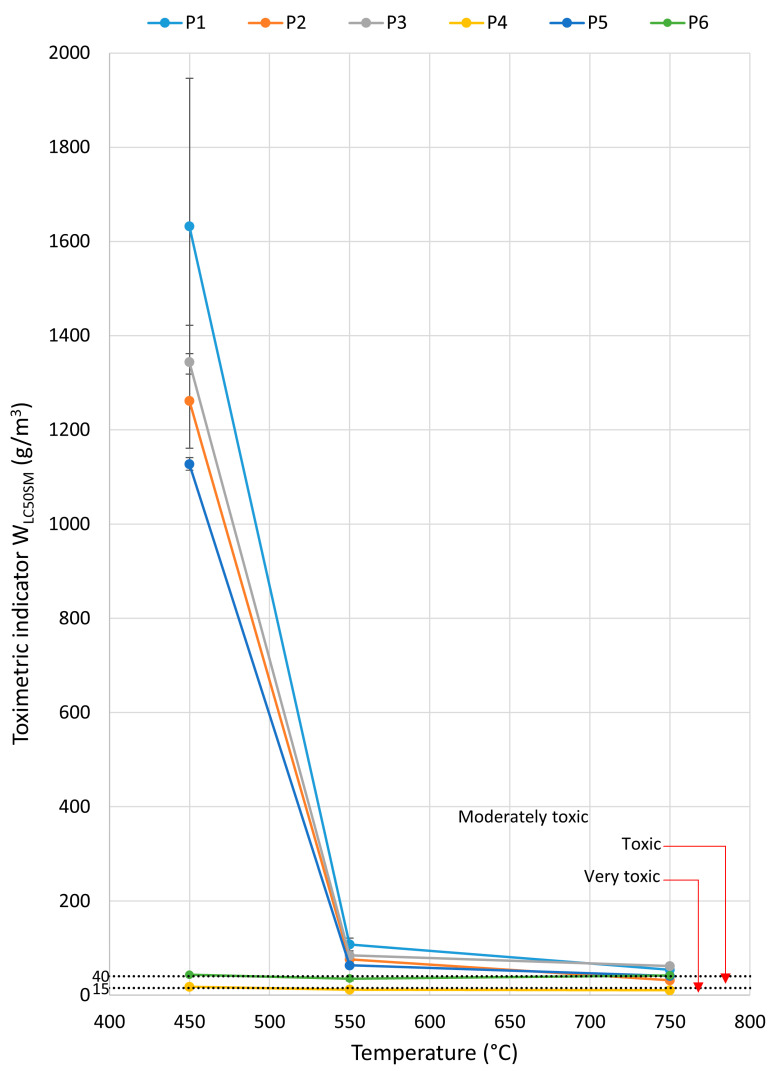
The relationship between the toxicometric indicator (WL_C50SM_) and the temperature for the tested belts.

Materials used in drive and toothed belts can pose a risk to human health and safety due to the release of toxic byproducts during thermal decomposition and combustion, including CO, CO_2_, HCN, SO_2_, NO, NO_2_, and HCl. They may affect safe evacuation conditions. The emission of toxic gases from materials burning at concentrations exceeding levels lethal to humans can cause threats to health and life, effectively preventing evacuation. The W_LC50M_ toxicometric indicator, which results from the W_LC50_ indicator of individual products of thermal decomposition and combustion for a given temperature, makes it possible to determine the effect of the thermal decomposition and combustion temperature on the toxic properties of the material under these conditions. The values of the toxicometric indicators (W_LC50M_) measured at 450, 550, and 750 °C for the tested toothed belts are presented in Figure 8. The toxicometric indicator (W_LC50M_) is low over the entire range of temperatures tested for the rubber matrix toothed belt samples (P4 and P6). However, for the polyurethane matrix belts (P1–P3, P5), the value of the toxicometric index (W_LC50M_) significantly decreases with the increasing combustion and thermal decomposition temperatures tested at 550 and 750 °C. The results of W_LC50M_ showed that belts made of polymeric materials have significantly lower toxic emissions at temperatures below 450 °C.

The P4 and P6 toothed belts, which have the least favourable toxicometric indicators (WLC50M), also exhibit the lowest mass loss and are the only ones to emit SO_2_ and HCl, with their WLC50SM indicator already being classified as toxic or highly toxic at 450 °C. A common feature distinguishing these belts from the others is their polyester cord. The lowest mass loss was observed in belts with a polyurethane matrix and a Kevlar cord, followed by belts with a polyurethane matrix and a steel cord. Belts with a polyurethane matrix and a Kevlar or steel cord are characterised by the emissions of NO, HCN, CO_2_, CO, and, in one case, NO_2_ (at 450 °C), along with a similar WLC50M characteristic as a function of temperature. It can be observed that the belts containing rubber (P4 and P6) emit sulphur-containing compounds, indicating that sulphur was used in the vulcanisation process of the belt. Steel cords themselves do not release toxic gases but can act as thermal conductors, accelerating the combustion process of surrounding materials. The combination of different materials (PU, rubber, Kevlar, steel, nylon) leads to complex chemical interactions during burning, sometimes producing even more hazardous byproducts than those found in single-material combustion.

**Figure 9:**
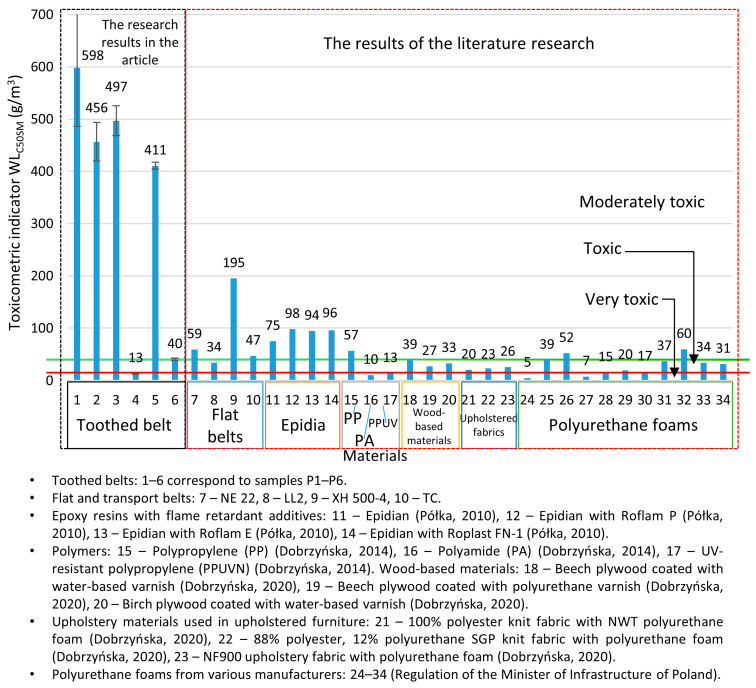
Toxicometric index (WL_C50SM_) values for various construction materials [61,62,63,64].

The obtained toxicometric indicator (WL_C50SM_) results were analysed in comparison with the classification criteria for the toxicity of the thermal decomposition and combustion products, as specified in PN-B-02855, and are illustrated in Figure 9. The obtained results of the toxicometric indicators (W_LC50SM_) were related to the classification criteria of toxicity of the products of thermal decomposition and combustion in accordance with PN-B-02855 and are shown in Figure 9. Most of the toothed belts are classified as moderately toxic material (P1–P3 and P5), and these belts contain a polyurethane matrix. A toothed belt (P6) in a rubber matrix with a polyurethane cord is classified as a toxic material. The most hazardous material under fire conditions is the P4-toothed belt with a rubber matrix and Kevlar cord; it is characterised as a very toxic material. In comparing toothed belts and flat belts (Figure 9), it can be seen that toothed belts are less toxic than flat belts. In particular, polyurethane matrix toothed belts are characterised as less toxic than polyurethane flat belts, as flat belts often have a multilayer structure consisting of polymers with different chemical compositions. It should be noted that, of the many materials tested in terms of the WL_C50SM_ index, most toothed belts are less harmful to humans and the environment than Epidian [61], polyamide PA [62], polypropylene PP [62], UV-resistant polypropylene: PPUVN [62], wood-based materials [63], upholstered fabric [63], and polyurethane foams [64]. Drive and transport belts are commonly used in machines and devices, where most components are made of metal and are, therefore, non-flammable. As a result, these belts can be considered a primary source of toxic emissions during a fire. However, other flammable elements within the machine’s operating environment cannot be ruled out. Combustible machine components, the surrounding equipment, and the transported materials all contribute to the overall fire hazard. The greatest concern arises from toxic substances, which may be restricted or prohibited by regulations, particularly when used indoors [64]. However, the presence of moderately toxic or toxic materials in a fire does not imply safety. The toxicometric indicator (WL_C50SM_) represents an average of the individual toxicometric values determined for all decomposition and combustion products at three different temperatures. However, an accurate assessment of toxic fire hazards should consider the specific quantities of the individual toxic gases released during each fire stage, with particular emphasis on the early phase, when effective evacuation is still possible. To properly evaluate the actual toxic risk in a fire scenario, toxicometric indicators (WL_C50SM_) should be carefully analysed. This issue becomes even more critical given that smoke inhalation and toxic fire byproducts account for 60–80% of fatalities in residential fires [65,66,67,68], despite regulations requiring that every enclosed space, such as buildings and transport vehicles, must ensure safe evacuation in the event of a fire. The results of the presented studies may help evaluate fires in conveyor belts and other equipment in technological processes equipped with strand transmissions. Air purification is recommended in places where the air was polluted due to the thermal decomposition or combustion of toothed belts.

When describing the issues of extinguishing fires involving toothed belts made of plastics, the following extinguishing agents can be used: AR-AFFF foaming agents, or extinguishing powder (ABC). A fire blanket is also a great solution, which allows for the suppression and isolation of the fire. The experience gained shows that water extinguishing agents can also be used to extinguish plastic fires, but only those that are supplied in a dispersed stream (e.g., water mist systems). Otherwise, droplets (burning drops) may occur, which may contribute to the formation of secondary fires. This is an interesting topic and may inspire us to expand research work in this area.

## 4. Conclusions

In conclusion, toothed belts are used in many machines and types of equipment, especially those requiring synchronised operation. They are usually made of composite materials with different matrixes (e.g., thermoplastic polyurethane, polyamide, rubber reinforced with a plasmid, and/or steel cord), which can pose a serious risk of toxic compounds under fire conditions. Belt designs and materials are being developed to improve the mechanical strength and reduce the energy consumption of gearboxes, but the risk of their use resulting from the behaviour of the belts during a fire has not been assessed. The release of toxic byproducts from the thermal decomposition and combustion of toothed belts can pose a severe threat in fire-prone environments, where these belts are utilised. The material classification based solely on the toxicometric indicator (W_LC50SM_) is insufficient for accurately assessing the actual fire hazard. However, its results make it possible to identify and eliminate the use of the materials that pose the most significant risk under fire conditions. During the thermal decomposition and combustion of toothed belts, CO, CO_2_, HCN, SO_2_, NO, NO_2_, and HCl emissions can occur. The least favourable toxicometric indices (WLC50SM) were observed in the P4 and P6 rubber matrix belts, which were classified as highly toxic (13 g/m^3^) and toxic (40 g/m^3^) materials, respectively. Additionally, these were the only belts that exhibited SO_2_ and HCl emissions. These belts are distinguished by the use of polyester cords. The polyurethane matrix belts (P1–P3, P5) were characterised as moderately toxic materials, with W_LC50SM_ ranging from 411 g/m^3^ to 598 g/m^3^. A relevant parameter for assessing the threat posed by toxic combustion products is the critical mass of the material, which defines the maximum amount that can be used in a given room without exceeding the concentration limits of the thermal decomposition and combustion products in the event of a fire. However, published studies indicate that toothed belts require improvements in their chemical composition to minimise their negative impact on human health and life during a fire, especially those containing a rubber matrix. Work should begin on replacing rubber in belt construction with materials such as polyurethane, polyamides, or silicone elastomers. Alternatively, additives can be incorporated into the rubber to reduce the toxicity of fire gases, including hydroxytetraphosphates (e.g., Roflam P, Roflam E), nanoadditives (e.g., nanoclays, carbon nanotubes), metal oxides (e.g., MgO, Al_2_O_3_, ZnO), and silicon compounds (e.g., polysiloxanes).

## Figures and Tables

**Table 1:** Comparison of identified substances in fumes after performing tests in accordance with PN-B-02855.

		Chemical Substances								
		SO_2_	NO_2_	NO	HCN	CO_2_	CO	HCl	HBr	HF
Toothed belt	P1	−	−	+	+	+	+	−	−	−
	P2	−	−	+	+	+	+	−	−	−
	P3	−	+	+	+	+	+	−	−	−
	P4	+	−	+	+	+	+	+	−	+
	P5	−	−	+	+	+	+	−	−	−
	P6	+	−	−	−	+	+	+	−	−

## Data Availability

The original contributions presented in this study are included in the article. Further inquiries can be directed to the corresponding author.
